# Therapeutic potential of tonsil-derived mesenchymal stem cells in dextran sulfate sodium-induced experimental murine colitis

**DOI:** 10.1371/journal.pone.0183141

**Published:** 2017-08-30

**Authors:** Yeonsil Yu, Eun Mi Song, Ko Eun Lee, Yang-Hee Joo, Seong-Eun Kim, Chang Mo Moon, Ha Yeong Kim, Sung-Ae Jung, Inho Jo

**Affiliations:** 1 Department of Molecular Medicine, College of Medicine, Ewha Womans University, Seoul, South Korea; 2 Department of Internal Medicine, College of Medicine, Ewha Womans University, Seoul, South Korea; Future University, EGYPT

## Abstract

The therapeutic potential of tonsil-derived mesenchymal stem cells (TMSC) prepared from human tonsillar tissue has been studied in animal models for several diseases such as hepatic injury, hypoparathyroidism, diabetes and muscle dystrophy. In this study, we examined the therapeutic effects of TMSC in a dextran sulfate sodium (DSS)-induced colitis model. TMSC were injected in DSS-induced colitis mice via intraperitoneal injection twice (TMSC[x2]) or four times (TMSC[x4]). Control mice were injected with either phosphate-buffered saline or human embryonic kidney 293 cells. Body weight, stool condition and disease activity index (DAI) were examined daily. Colon length, histologic grading, and mRNA expression of pro-inflammatory cytokines, *interleukin 1β* (*IL-1β*), *IL-6*, *IL-17* and *tumor necrosis factor α*, and anti-inflammatory cytokines, *IL-10*, *IL-11* and *IL*-*13*, were also measured. Our results showed a significant improvement in survival rates and body weight gain in colitis mice injected with TMSC[x2] or TMSC[x4]. Injection with TMSC also significantly decreased DAI scores throughout the experimental period; at the end of experiment, almost complete reversal of DAI scores to normal was found in colitis mice treated with TMSC[x4]. Colon length was also significantly recovered in colitis mice treated with TMSC[x4]. However, histopathological alterations induced by DSS treatment were not apparently improved by injection with TMSC. Finally, treatment with TMSC[x4] significantly reversed the mRNA levels of *IL-1β* and *IL-6*, although expression of all pro-inflammatory cytokines tested was induced in colitis mice. Under our experimental conditions, however, no apparent alterations in the mRNA levels of all the anti-inflammatory cytokines tested were found. In conclusion, our findings demonstrate that multiple injections with TMSC produced a therapeutic effect in a mouse model of DSS-induced colitis.

## Introduction

Inflammatory bowel disease (IBD) manifests as chronic inflammation of the colon and small intestine. Recently, the incidence of IBD including ulcerative colitis and Crohn’s disease, which can lead to life-threatening problems, has rapidly increased, and is thus a growing public health concern [1, 2]. IBD is caused by a variety of risk factors including genetic predisposition, environmental factors and food habits [3]; however, the exact incidental mechanisms of IBD development remain largely unknown. Although anti-inflammatory and immunosuppressant drugs such as 6-mercaptopurines, cyclosporine, anti-tumor necrosis factor α (TNF-α) antibody, 5-aminosalicylic acid and corticosteroids are currently available for IBD treatment [4–6], it is difficult to achieve an ideal therapy on IBD. Severe side effects are frequent in IBD treatment, and thus development of more effective drugs for IBD treatment remains a challenge.

Previously, a cell-based therapeutic strategy using mesenchymal stem cells (MSC) including bone marrow-derived MSC (BM-MSC) and adipose tissue-derived MSC (A-MSC) was proposed for the treatment of inflammatory diseases such as graft-versus-host disease [7], arthritis [8, 9], and pancreatitis [10]. The mechanism underlying MSC-mediated therapeutic potential in inflammatory diseases is related to the immunosuppressive properties of MSC. MSC are capable of reducing the cells comprising a network of immune system such as dendritic cells, NK cells and T-cells [11–13], and also of elevating secretion of anti-inflammatory cytokines such as interleukin 10 (IL-10) and prostaglandin E2 (PGE2) [14]. These findings suggest that IBD is a disease that could be effectively treated using MSC therapy [15, 16]. The beneficial therapeutic effects of MSC such as BM-MSC and A-MSC on a DSS-induced murine IBD model, or more specifically, a colitis model, have been reported [16, 17].

Recently, we established tonsil-derived MSC (TMSC) and have shown several advantages in terms of clinical applications compared to other MSC. For example, TMSC can be obtained noninvasively from “waste tissue,” which is easily removed by surgical tonsillectomy. They proliferate at a faster rate than BM-MSC [18, 19]. Short tandem repeat analysis also revealed that TMSC from three independent donors form a chimera when the cells are mixed-cultured, indicating the feasibility of using TMSC for cell banking in future clinical settings [20]. Like other MSC, TMSC have adipogenic, osteogenic and chondrogenic differentiation potential. Furthermore, these cells can easily differentiate into various types of cells such as tenocyte, muscle, and peripheral nerve [21–23]. Interestingly, TMSC can also differentiate into endodermal origin cells, such as parathyroid cells [24, 25]. In addition to those distinct characteristics, TMSC exhibit immune regulatory activity like other MSC. Previously, TMSC have been shown to have an immunomodulatory effect on allergic rhinitis in mice via both the reduction of Th2 cytokines and ovalbumin-specific immunoglobulin E secretion from B cells [26]. In this report, the authors suggested the inhibition of innate cytokines IL-25 and IL-33 as the underlying mechanism. Previously, we also reported that TMSC alleviate concanavalin A-induced acute hepatitis, indicating their role in the treatment of immune-mediated liver disease [27].

## Material and methods

### Isolation and expansion of TMSC

Informed consent was obtained from all patients’ legal guardians and the study was approved by the Ewha Womans University Medical Center institutional review board (ECT-11-53-02). Written consent for using of tonsils, study, and publication was obtained from each of the included patients’ legal guardians. TMSC were isolated and cultured as described [19]. Briefly, the tonsils were obtained during tonsillectomy from patients younger than 10 years old. The tonsillar tissues were minced and digested in Roswell Park Memorial Institute medium 1640 (RPMI-1640) (Invitrogen, Carlsbad, CA, USA) containing 210 U/mL collagenase type I (Invitrogen) and 10 g/mL DNase (Sigma-Aldrich, St. Louis, MO, USA) for 30 min at 37°C. The digested tissue was washed with Dulbecco’s modified Eagle’s medium-high glucose (DMEM-HG) (Welgene, Daegu, South Korea) including 20% fetal bovine serum (FBS) (Invitrogen), and washed again with DMEM-HG with 10% FBS. Mononuclear cells were isolated by Ficoll-Paque (GE Healthcare, Little Chalfont, UK) density gradient centrifugation of the washed tonsil tissues. Isolated cells were then plated in the cell culture plates and non-adherent cells were removed after a 48-h seeding. The adherent cells were further cultured for 2 weeks, and expanded by passaging. These expanded cells are hereafter referred to as TMSC, and were stored in liquid nitrogen for future use.

### Surface immunophenotypic assessment of TMSC

To perform cell surface immunophenotypic characterization, TMSC (5 x 10^4^ to 1 x 10^5^ cells) were collected in a round-bottom tube and washed with phosphate-buffered saline (PBS) with 0.5% bovine serum albumin (BSA) and 0.01% NaN_3_. The cells were incubated for 30 min at 4°C with fluorescein isothiocyanate (FITC)-labeled antibodies against human CD34, CD45, CD90 and CD105 (BD Biosciences, San Jose, CA, USA) and phycoerythrin (PE)-labeled antibodies against human CD14 and CD73 (BD Biosciences). For the negative control, the respective isotype antibodies (IgG1-FITC for CD45 and CD90, IgG2-FITC for CD34 and CD105, IgG1-PE for CD73, IgG2-PE for CD14; BD Biosciences) were used. Cell surface immunophenotypic characterization was performed and results were analyzed using the BD FACS Calibur flow cytometer and BD Cell Quest^TM^ Pro Software (BD Biosciences), respectively.

### Adipogenic, osteogenic and chondrogenic differentiation of TMSC

To measure the mesodermal differentiation capacity of TMSC, TMSC (5 x 10^4^ cells) were seeded in 24-well cell culture plates. After 24 h of seeding, the cells were incubated in commercially available adipogenic, osteogenic and chondrogenic differentiation media (Invitrogen) as previously described [19]. The differentiation media was changed every 3 or 4 days for 2 weeks. After induction of differentiation, the cells were washed twice with PBS, fixed in 4% paraformaldehyde for 15 min at room temperature, and washed again with PBS. Thereafter, cells were stained with 2% Oil red O staining solution (Sigma-Aldrich) for adipogenic differentiation, with 2% Alizarin red S solution (Sigma-Aldrich) for osteogenic differentiation, or 1% Alcian blue staining solution (Sigma-Aldrich) for chondrogenic differentiation. After the cells were rinsed, the adipogenic, osteogenic and chondrogenic cells were visualized under a phase-contrast microscope.

### *In vitro* immune suppression assay

For the *in vitro* immune suppression assay, murine splenocytes were used as responder cells and phorbol 12-myristate 13-acetate (PMA) (Sigma-Aldrich) as a mitogen. Splenocytes (2 x 10^6^ cells) were first pre-stimulated with 200 ng/ml of PMA and then co-cultured for 24 h by adding to TMSC (3 x 10^4^ cells) pre-adhered in a 96-well plate (BD Bioscience). After co-culture, the proliferation of splenocytes was measured using a 5-bromo-2′-deoxyuridine (BrdU) cell proliferation assay kit (Cell Signaling Technology, Boston, MA, USA) as previously described [28]. Briefly, splenocytes were incubated with exogenous BrdU for 3 h and centrifuged. The cells were then fixed with fixing solution for 30 min. The incorporated BrdU was probed using an anti-BrdU antibody, followed by the corresponding HRP-conjugated secondary antibody solution. Cells were washed, and absorbance was measured at 450 nm.

### Animals and development of a chronic colitis mouse model

**Fig 1:**
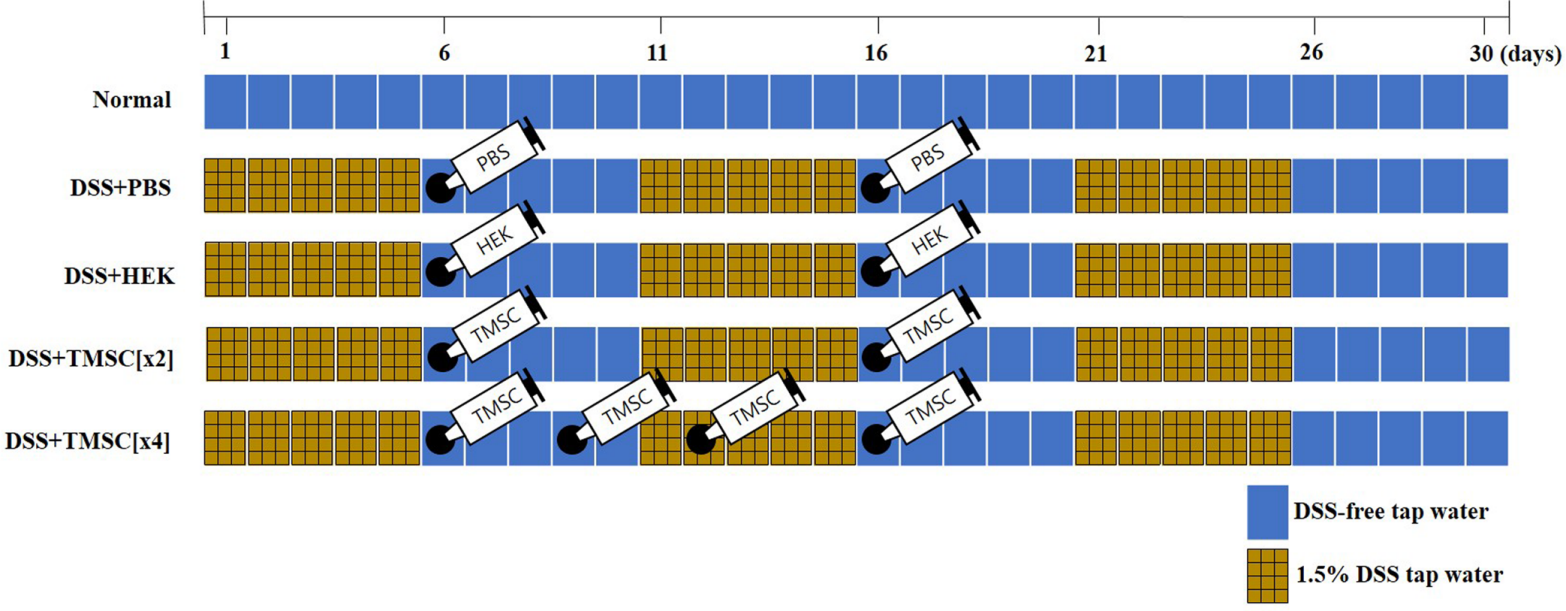
Experimental design. Mice (n = 45) were randomly assigned to five groups (n = 9 per group). Normal mice received only tap water and no injections. For induction of the chronic colitis mouse model, mice received tap water with 1.5% DSS (1.5% DSS tap water) three times for 5 days on days 1, 11, and 21. Mice in the DSS+PBS group were injected with 500 μL PBS on days 6 and 16. Mice in the DSS+HEK group were injected with HEK293 cells (1 x 10^6^ cells/mouse/day) on days 6 and 16. Mice in the DSS+TMSC[x2] group were injected with TMSC (1 x 10^6^ cells/mouse/day) on days 6 and 16. Mice in the DSS+TMSC[x4] group were injected with TMSC (1 x 10^6^ cells/mouse/day) on days 6, 9, 12, and 16.

Seven-week-old C57BL/6 male mice, with an average weight of 18–25 g, were purchased from Orient Bio Co., Ltd. (Orient Bio Co., Ltd., Gyeonggi, Korea). Mice were bred and maintained at the animal facility of the Medical School of Ewha Womans University, Seoul, South Korea. Mice were housed in a controlled environment in collective cages, containing three mice each, at 23±2°C and 50% humidity under a 12-h light/dark cycle with free access to food and water. Mice were allowed to acclimate to these conditions for at least 7 days before the experiment. The experimental protocol was reviewed and approved by the ethics committee for animal research of Ewha Womans University (based on the policies of the Institutional Animal Care and Use Committee) (ESM 14–0262). Institutional guidelines for animal care and use were followed throughout the experiments. Animals were monitored daily for signs of distress or advanced colitis. Colitis was induced in C57BL/6 mice by oral administration of dextran sulfate sodium (DSS) (molecular weight 36–50 kDa, MP biochemical, Irvine, CA, USA) as previously described [29, 30]. Briefly, 1.5% DSS was freshly prepared whenever DSS administration began. Mice were exposed to 3 cycles of five-day DSS administration (DSS tap water), with five-day DSS-free tap water in between. We also used PBS or human embryonic kidney 293 (HEK293) cells for sham control (Fig 1). The average amount of DSS tap water taken was recorded for the amount of DSS administered. Humane endpoints were in place but were not used because the animals died when we were not in the animal facility even though we checked daily for signs of distress or advanced colitis.

### *In vivo* experimental design

Male mice (n = 45) were randomly assigned to the following five groups (n = 9 per each group): (1) **Normal**, mice received no DSS tap water intake; (2) **DSS+PBS**, mice received 1.5% DSS tap water three times on days 1, 11, and 21. DSS tap water was given for five days each time. Mice received intraperitoneal (IP) injections of 500 μL PBS on days 6 and 16; (3) **DSS+HEK**, mice received the same as DSS+PBS mice, except that 500 μL PBS was replaced with HEK293 cells (1 x 10^6^ cells/mouse/day); (4) **DSS+TMSC[x2]**, mice received the same as DSS+PBS mice except for replacing 500 μL PBS with TMSC (1 x 10^6^ cells/mouse/day) twice on days 6 and 16; (5) **DSS+TMSC[x4]**, mice received the same as DSS+PBS except for replacing 500 μL PBS with TMSC (1 x 10^6^ cells/mouse/day) four times on days 6, 9, 12, and 16. For TMSC or HEK293 cell injection, the indicated numbers of cells were re-suspended in PBS and then gently administered via an intraperitoneal route using a 1-ml insulin syringe twice or four times. Mice were monitored by daily DAI scoring, and their body weight and stool conditions were recorded. At days 6, 18, and 31 during the experimental period, mice were mildly anesthetized by intraperitoneal injection of 24 mg/kg of zoletil-100 (Virbac, Carros, France) and 2 mg/kg of Rompun (Bayer, Leverkusen, Germany) mixture and were then sacrificed. All experiments were independently repeated at least three or four times.

### Disease activity index (DAI) scoring

Mice were monitored daily by recording body weight and stool conditions such as stool consistency and bleeding, which were subjectively scored by two independent researchers from grade 0 to 4 according to the following DAI scoring system (Table 1)[31].

**Table 1:** Disease activity index (DAI) scoring.

Score	Weight loss	Stool consistency	Occult/gross rectal bleeding
0	None	Normal	Normal
1	1–5%	-	-
2	5–10%	Loose	Hemoccult
3	10–20%	-	-
4	>20%	Diarrhea	Gross rectal bleeding

### Histopathological examinations

#### Sample preparation

Colons were surgically removed and subjected to length measurement. In some experiments, 1-cm colon tissues cut from the distal end were collected for histopathological examinations. Other remaining tissues were used for RNA preparation.

#### Hematoxylin and eosin (H & E) stain and histologic scoring

Distal colon tissues were fixed with formalin overnight and embedded in paraffin. Histological analysis was performed on the 4-μm paraffin sections as previously described [32]. Briefly, tissue sections were mounted on slides, deparaffinized, rehydrated, and stained with hematoxylin. After washing with tap water, the sections were stained with eosin for 2 min. The sections were microscopically examined for histopathological changes using the following scoring system (Table 2).

**Table 2:** Histologic colitis scoring system [31].

Features	Score	Description
Inflammation severity	**0**	None
	**1**	Mild
	**2**	Moderate
	**3**	Severe
Inflammation extent	**0**	None
	**1**	Mucosa
	**2**	Submucosa
	**3**	Transmural
Crypt damage	**0**	None
	**1**	Basal 1/3 damage
	**2**	Basal 2/3 damage
	**3**	Crypt lost; surface epithelium present
	**4**	Crypt and surface epithelium lost
Percent involvement	**0**	0%
	**1**	1–25%
	**2**	26–50%
	**3**	51–75%
	**4**	76–100%

#### Quantitative real-time polymerase chain reaction (real time PCR)

Total RNA was extracted using easy-BLUE^TM^ total RNA extraction kit (cat. no. 17061, Intron biotechnology, Gyeonggi, Korea) as described in the manufacturer’s instructions. Briefly, corresponding frozen colonic tissues were first homogenized by sonication in 1 mL of easy-BLUE^TM^ reagent and then chloroform (Sigma) was added. After centrifugation, the supernatants were mixed with isopropanol (Sigma), and centrifuged to obtain RNA pellets. RNA pellets were then washed with 75% ethanol. Reverse transcription was performed with Moloney murine leukemia virus reverse transcriptase (Promega, Fitchburg, WI, USA) with 2 μg of RNA. Real time-PCR was performed using 2x Power SYBR Green PCR Master mix (Applied Biosystems, Waltham, MA, USA) and primers for pro-inflammatory cytokine genes, *IL-1 β*, *IL-6*, *IL-17* and *TNF-α*, or anti-inflammatory cytokine genes, *IL-10*, *IL-11* and *IL-13*. Primer for *glyceraldehyde 3-phosphate dehydrogenase* (*GAPDH*) was also used for a loading control. All the primers were produced by Macrogen (Macrogen, Seoul, Korea), and their sequences are listed in Table 3. RT-PCR analysis was performed using the 7000 Real time-PCR system (Applied Biosystems).

**Table 3:** Primer sequences of polymerase chain reaction.

Gene*		Primer sequences
*IL-1β*	Forward	5'-GAGCCCATCCTCTGTGACTC-3'
	Reverse	5'-TCCATTGAGGTGGAGAGCTT-3'
*IL-6*	Forward	5’-ATGAAGTTCCTCTCTGCAAGAGACT-3’
	Reverse	5’-CACTAGGTTTGCCGAGTAGATCTC-3’
*IL-17*	Forward	5'-TCCCTCTGTGATCTGGGAAG-3'
	Reverse	5'-CTCGACCCTGAAAGTGAAGG-3'
*TNF-α*	Forward	5’-ATGAGCACAGAAAGCATGATC-3’
	Reverse	5’-TACAGGCTTGTCACTCGAATT-3’
*IL-10*	Forward	5’-CAGCCGGGAAGACAATAACT-3’
	Reverse	5’-TCATTTCCGATAAGGCTTGG-3
*IL-11*	Forward	5'-CTGCACAGATGAGAGACAAATTCC-3'
	Reverse	5'-GAAGCTGCAAAGATCCCAATG-3'
*IL-13*	Forward	5'-GGAGCTGAGCAACATCACAC-3'
	Reverse	5'-GGTCCTGTAGATGGCATTGCA-3'
*GAPDH*	Forward	5’-TGATGACATCAAGAAGGTGGTGAAG-3’
	Reverse	5’-TCCTTGGAGGCCATGTGGGCCAT-3’

^*^IL-1β, interleukin 1β; *IL-6*, interleukin 6; *IL-17*, interleukin 17; *TNF-α*, tumor necrosis factor α; *IL-10*, interleukin 10; *IL-11*, interleukin 11; *IL-13*, interleukin 13; *GAPDH*, glyceraldehyde 3-phosphate dehydrogenase

### Statistical analysis

Data are presented as mean ± standard deviation (SD). Differences of long-term survival rates were compared by *χ*^2^ test. Differences between the two groups (data at day 6) were evaluated by *t*-test (*P<0.05, **P<0.01, and ***P<0.001). Differences among more than the two groups (data at days 18 and 31) were analyzed by Kruskal-Wallis test followed by Dunn’s multiple comparison test using GraphPad Prism software ver. 6 (GraphPad Software, Inc., La Jolla, CA, USA). Different letters indicate significant differences among experimental groups (*P*<0.05).

## Results

### Surface immunophenotypic characteristics and differentiation potentials of TMSC

**Fig 2:**
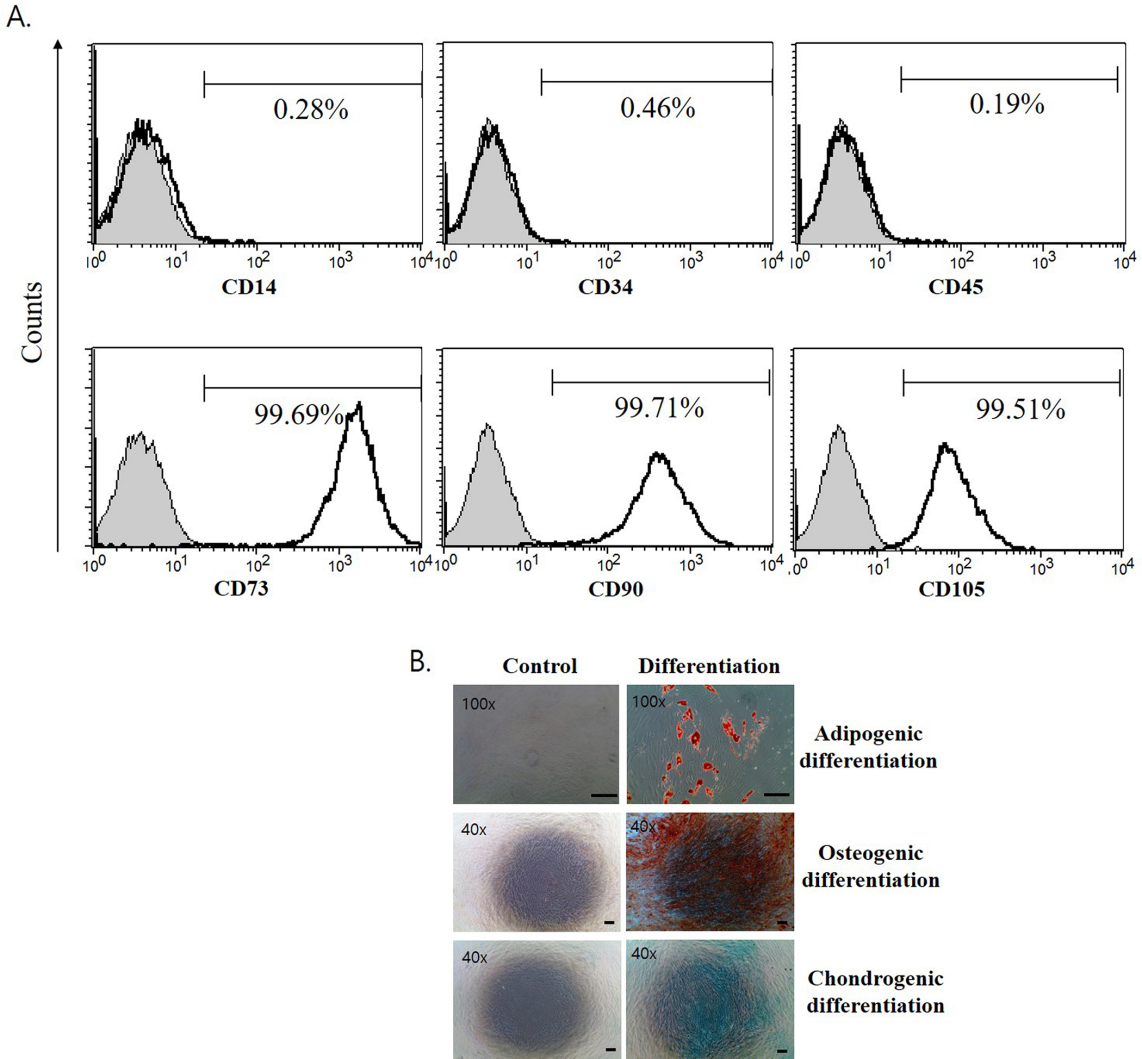
Immunophenotypes and differentiation capacity of the cell surface of TMSC. (A) Representative flow cytometry analysis of TMSC are shown. The cells were labeled with antibodies against human antigens, CD14, CD34, CD45, CD73, CD90, and CD105. The shaded histogram indicates isotype control and the open histogram represents positive reactivity with the indicated monoclonal antibody. (B) TMSC were induced to differentiate into adipogenic, osteogenic, and chondrogenic lineages for 3 weeks using the appropriate differentiation medium. For a control experiment, TMSC were cultured in the absence of differentiation medium. The level of adipogenesis, osteogenesis, and chondrogenesis was assessed by staining with Oil red O, Alizarin red S, and Alcian blue, respectively. Scale bar: 200 μm.

As shown in Fig 2A, the immunophenotypic surface marker assay of TMSC revealed a typical MSC surface phenotype; hematopoietic cells markers such as CD14, CD34 and CD45 were negative, while common MSC markers such as CD73, CD90 and CD105 were positive. Furthermore, cell-specific staining assays for differentiation into adipocytes, chondrocytes and osteoblasts showed that TMSC could differentiate into three different cell types, adipocytes, chondrocytes and osteoblasts, respectively, through induction using commercially-available differentiation media (Fig 2B).

### TMSC suppress splenocyte proliferation induced by PMA

**Fig 3:**
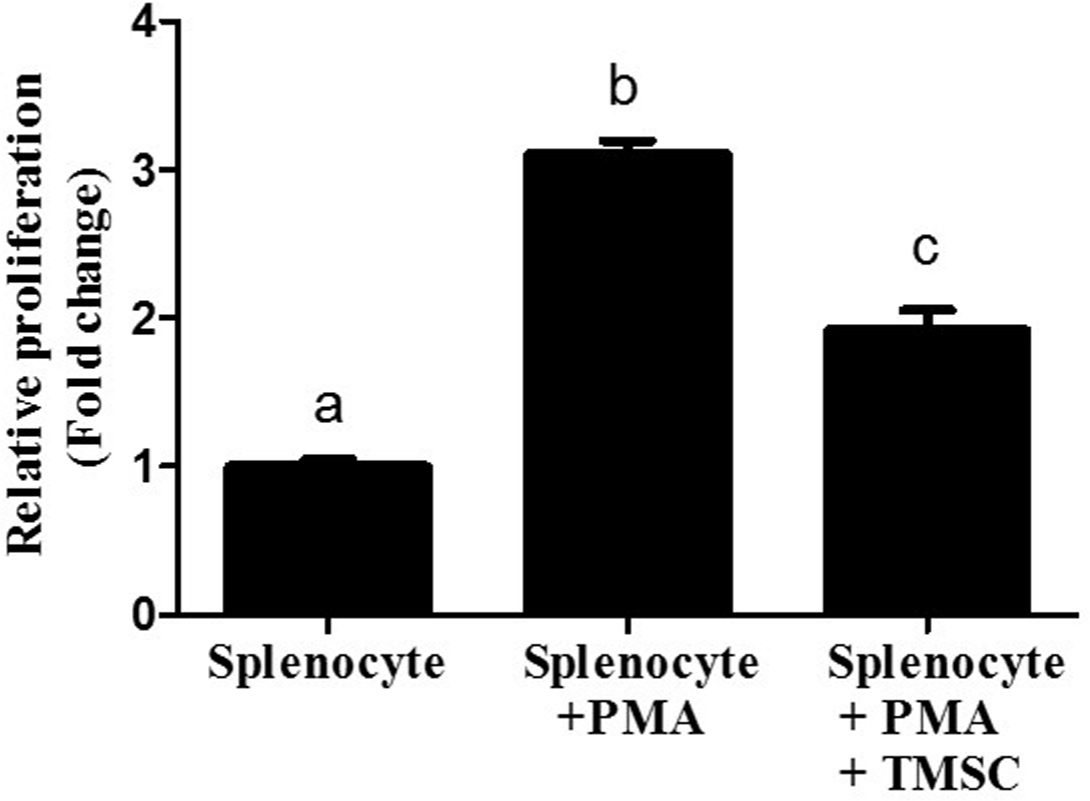
TMSC demonstrate immune regulatory activity *in vitro*. For the immune suppression assay, murine splenocytes, as responder cells, were stimulated with PMA. After the addition of TMSC, we found that PMA-stimulated proliferation of splenocytes was significantly reduced (n = 3). Different letters indicate significant differences among experimental groups (*P* < 0.05).

Co-culture with TMSC clearly inhibited the PMA-stimulated proliferation of splenocytes by ~60% compared with the control, suggesting the *in vitro* immunosuppressive capability of TMSC (Fig 3).

### TMSC injection improves survival rates and inhibits weight loss in chronic experimental colitis mice

Overall, the survival rate of colitis mice was significantly lower (~78% compared to the control) at the end of the experiment (day 31) (Table 4), which was improved to that of the normal mice by treatment with TMSC[x4]. TMSC[x2] injection also significantly improved the survival rate to ~89% of the control.

**Table 4:** Number of long-term survivors (%) by treatments.

Day	Number of survivors (%)					*P*
	Normal	DSS+PBS	DSS+HEK	DSS+ TMSC[x2]	DSS+ TMSC[x4]	
**10**	36 (100)	35 (97.2)	36 (100)	35 (97.2)	36 (100)	0.55
**15**	36 (100)	33 (91.6)	36 (100)	35 (97.2)	36 (100)	0.069
**20**	36 (100)	30 (83.3)	29 (80.5)	35 (97.2)	36 (100)	0.0012
**25**	36 (100)	28 (77.7)	29 (80.5)	34 (94.4)	36 (100)	0.0003
**31**	36 (100)	28 (77.7)	29 (80.5)	32 (88.8)	36 (100)	0.0022

**Fig 4:**
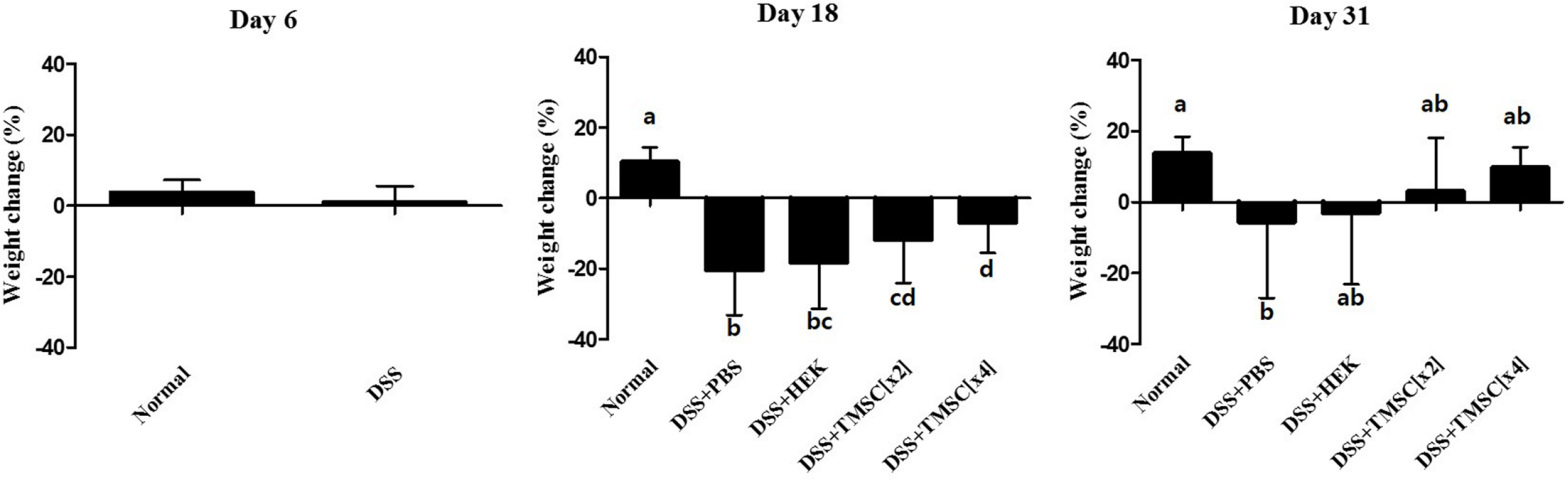
TMSC injection improves weight maintenance. Body weight was measured daily and expressed as body weight change (%) at days 0, 6, 18, and 31.

As shown in Fig 4, control mice showed a weight gain of ~15% throughout the experimental period. Instead, DSS-treated colitis mice had significant weight loss of ~20% at day 18, but this weight loss was diminished by ~5.5% of the initial weight at the end of the experiment. Treatment with TMSC[x2] or TMSC[x4] minimized DSS-induced weight loss at day 31; instead of weight loss, it rather showed a weight gain of ~3% or ~10%, respectively. HEK293 cells injection failed to inhibit DSS-induced weight loss.

### TMSC injection decreases DAI scores of experimental colitis mice

**Fig 5:**
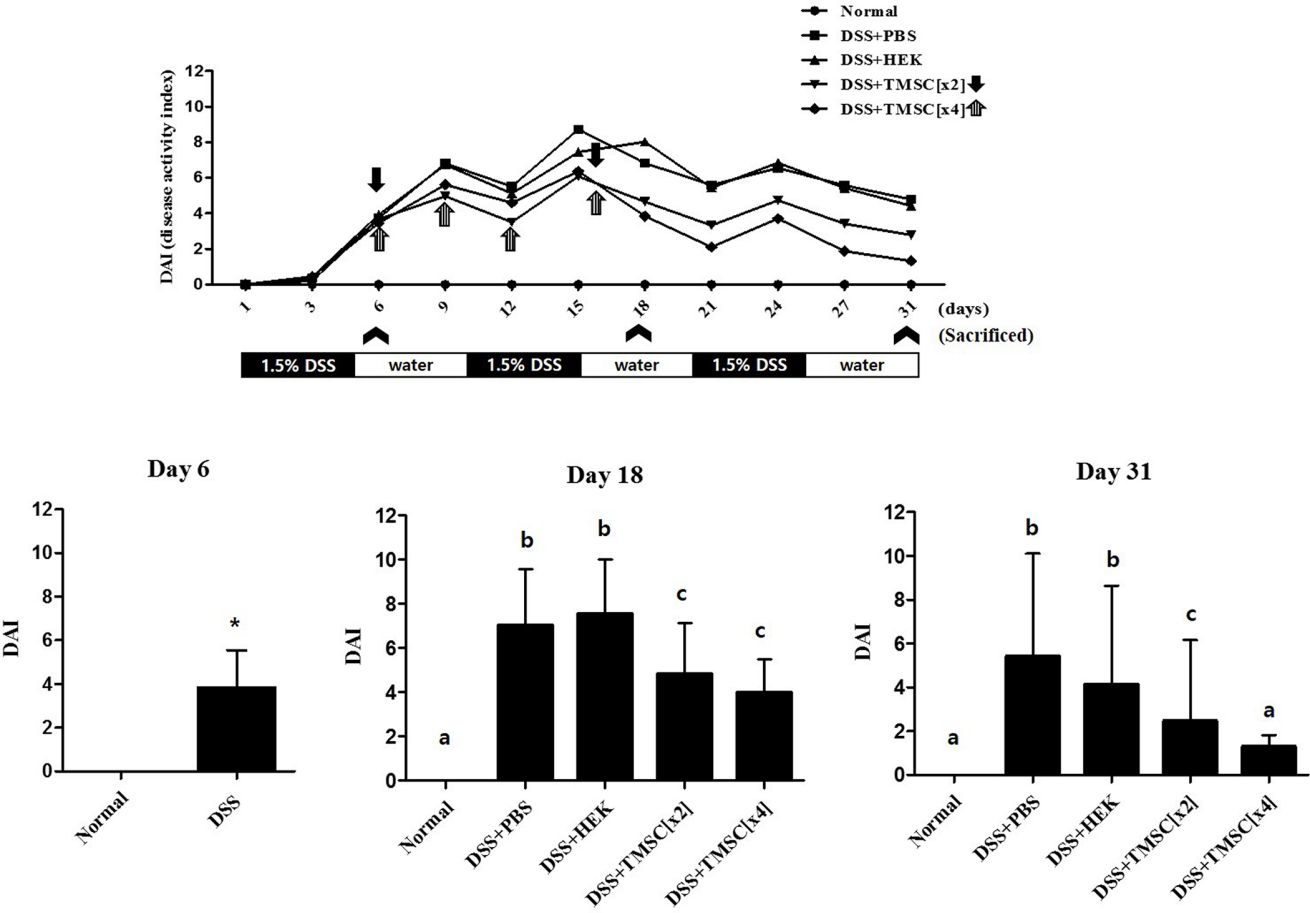
TMSC injection ameliorates inflammatory symptoms assessed by disease activity index (DAI) in DSS-induced colitis mice. DAI scores based on the presence of loose stools, fecal blood and weight loss were determined daily, and DAI scores at days 6, 18, and 31 are shown in the bar graphs.

Except for normal mice, all of the DSS-treated mice had diarrhea at day 3 that was aggravated until day 6. As shown in Fig 5, DAI scores in all groups generally showed an upward trend until day 15, despite some variation, and thereafter displayed a downward trend until the end of the experiment. The DAI scores of colitis mice injected with TMSC[x2] or TMSC[x4] were always lower than those injected with PBS or HEK293 cells at each time point; treatment with TMSC[x4] further decreased DAI scores compared to treatment with TMSC[x2], suggesting an injection number-dependent recovery efficiency. In particular, we found no statistically significant differences in DAI scores between TMSC[x4] treatment and the control at day 31.

### TMSC treatment inhibits DSS-induced colon shortening

**Fig 6:**
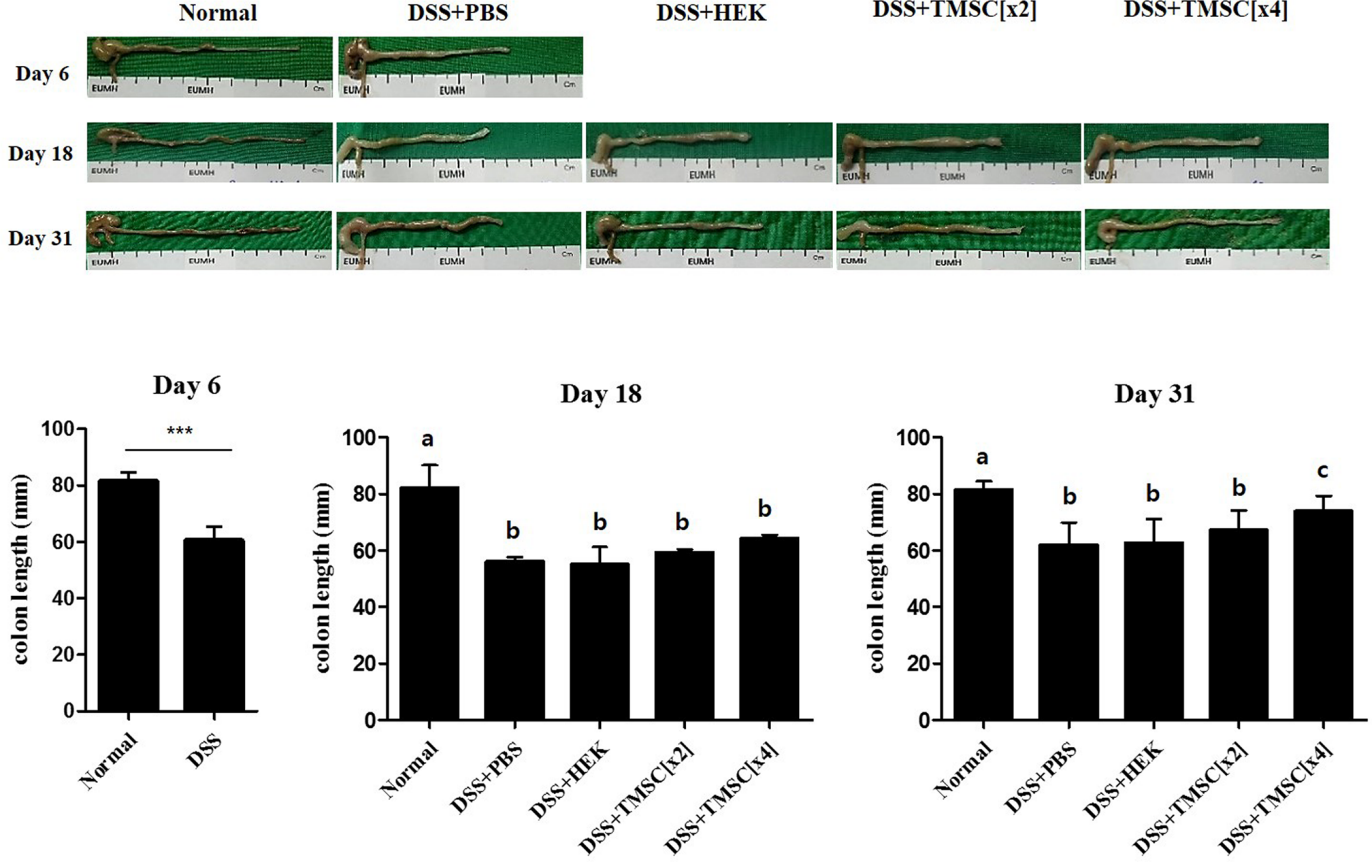
TMSC injection inhibits DSS-induced colon shortening. After sacrificing the mice, colons were dissected, and images of representative colon were obtained at days 6, 18, and 31. Mice in the DSS-treated group showed shortening of colon length at day 6, which was significantly inhibited by TMSC[x4] on day 31.

Colon length in control mice was 81.6 ±2.89 mm, which was significantly and rapidly shortened by ~26% on day 6 by DSS; this shortening was not altered during the rest of the experimental period. Treatments with TMSC appeared to inhibit colon shortening by DSS, but these results were only statistically significant with TMSC[x4] treatment on day 31 (Fig 6); DSS-induced colon shortening significantly decreased to ~9% in mice treated with TMSC[x4] compared with the control group.

### TMSC injection does not inhibit histopathological alterations in the distal colon in the chronic colitis mouse model

**Fig 7:**
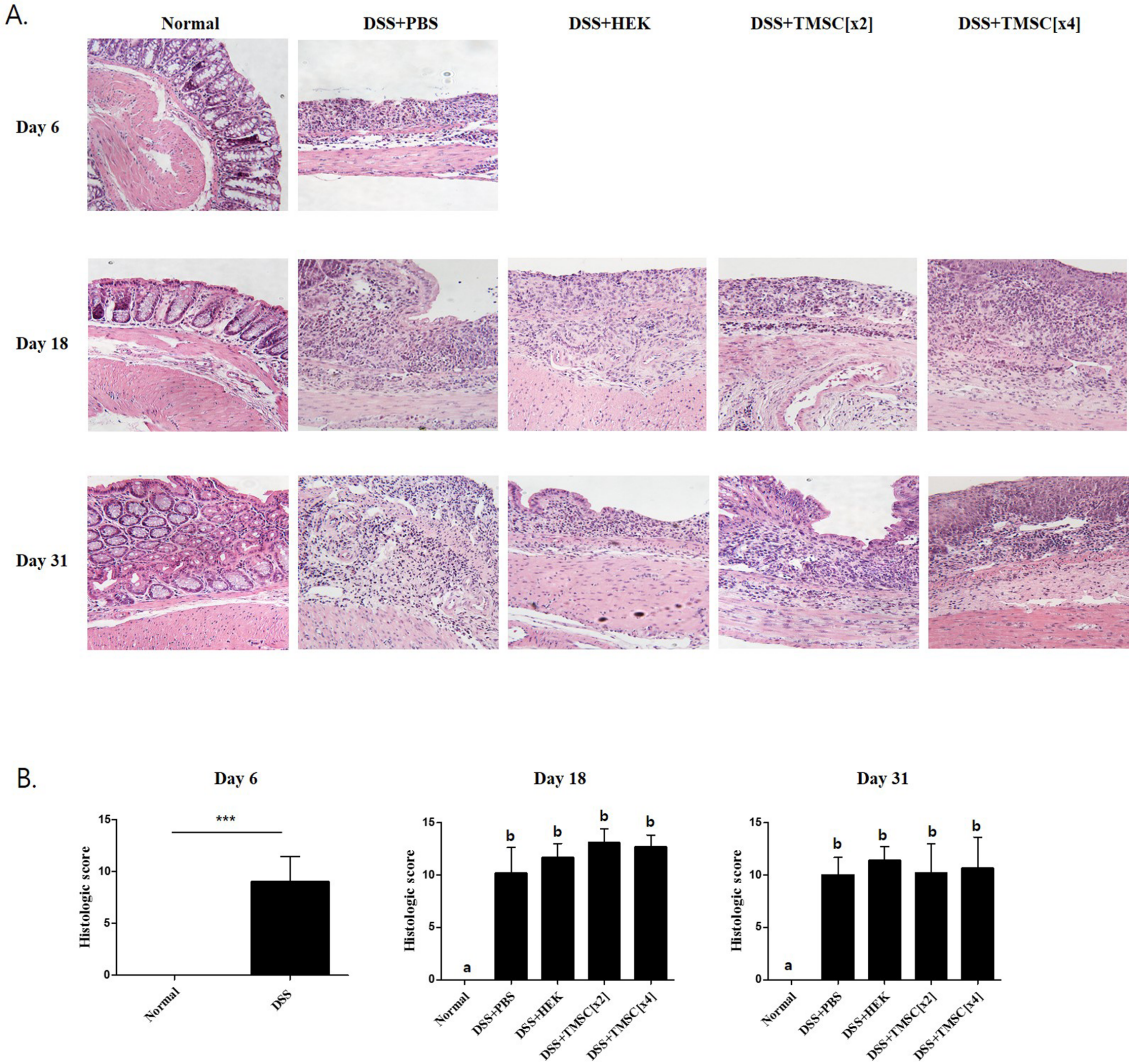
DSS-related histologic findings were not affected by TMSC[x4] treatment. Representative histological sections of mouse colons stained with hematoxylin and eosin (H&E) are shown, indicating no significant differences. Original magnification 200x.

Because the histopathological damage by DSS is predominantly found in the distal colon [33], we examined the distal colon tissue, which is located 1 cm from the anal verge. As shown in Fig 7A, colitis mice exhibited significant alterations in colon structure, such as decreases in goblet cells, infiltration of mononuclear cells, and induction of aberrant crypts. Like PBS and HEK293 cells, however, we failed to improve these alterations when TMSC[x2] and TMSC[x4] were administered. Using a histologic scoring system [31], we analyzed quantitatively histopathological alterations by colitis model. As expected, the histologic scores in DSS treatment group on days 18 and 30 were significantly higher than that of control mice; 10.1±2.4 and 10.0±1.6 versus 0, respectively (Fig 7B). However, these scores in DSS treatment group were not reduced by TMSC injection, although slightly, but not statistically significantly, higher scores in TMSC group were found on day 18; these were 10.1±2.4, 11.6±1.3, 13.0±1.3 and 12.6±1.1 in DSS+PBS, DSS+HEK, TMSC[x2] and TMSC[x4] group, respectively. Likewise, we also found almost the same scores on day 31 among different treatment groups, showing 10.0±1.6, 11.4±1.3, 10.0±2.8 and 10.6±2.9, respectively.

### TMSC treatment ameliorates IL-1β and IL-6 mRNA production in chronic colitis mice

**Fig 8:**
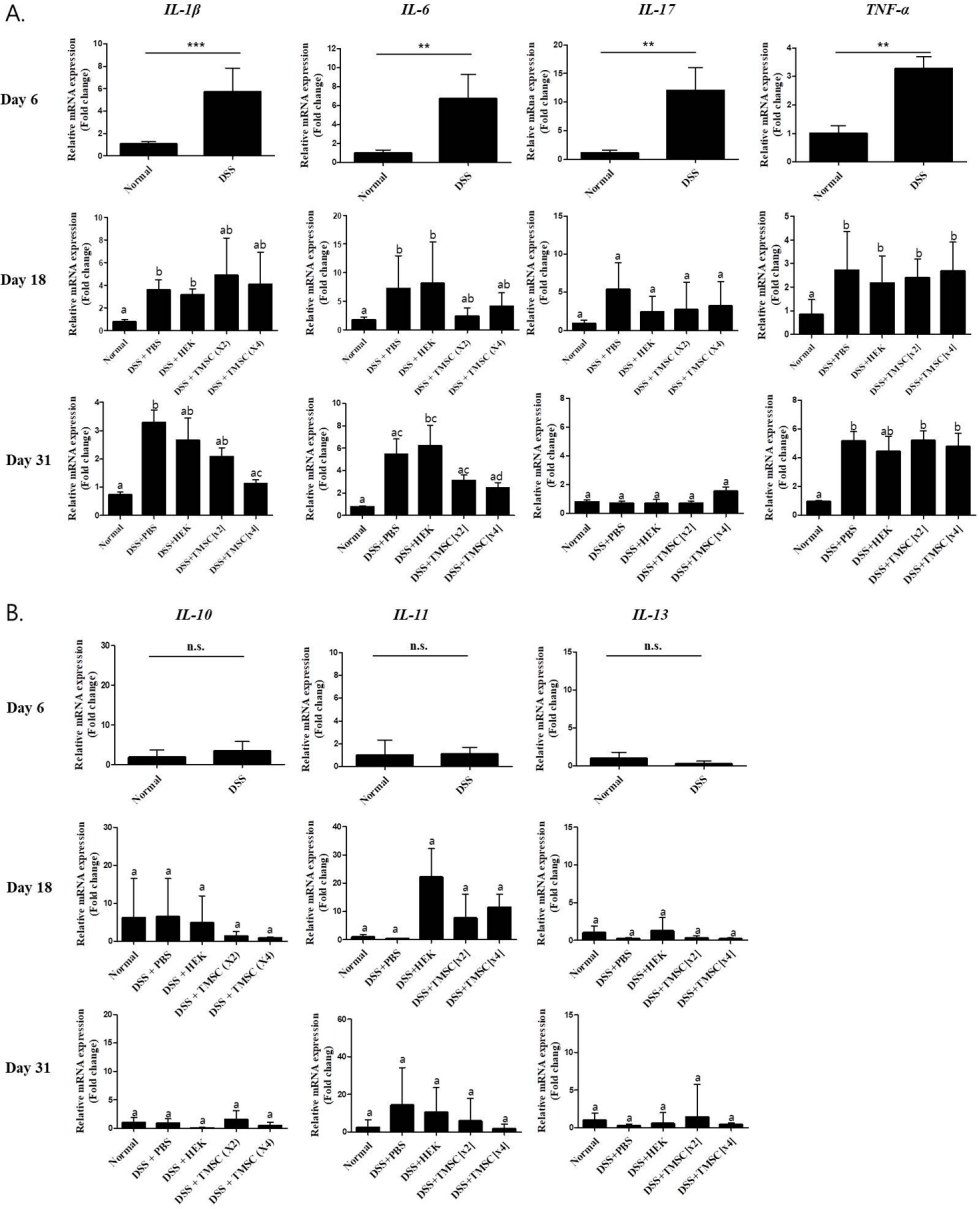
TMSC treatment ameliorates *IL-1β* and *IL-6* mRNA increases in chronic colitis mice. Colonic tissues were first homogenized by sonication. For measurement of cytokine levels, real-time PCR was performed using primers for pro-inflammatory cytokines (*IL-1β*, *IL-6*, *IL-17*, *TNF-α*)(A), and anti-inflammatory cytokines (*IL-10*, *IL-11*, *IL-13*)(B). *GAPDH* mRNA was used as a loading control. The mRNA levels of all the pro-inflammatory cytokines tested were significantly increased by DSS treatment. TMSC injection inhibited *IL-1β* and *IL-6* mRNA increased by DSS.

As shown in Fig 8A, significantly higher levels of mRNAs of pro-inflammatory cytokines, *IL-1β*, *IL-6*, *IL-17* and *TNF-α*, were found as early as day 6 in our colitis model than control group, showing ~5.7, ~6.7, ~12.0 and ~3.2 fold increase, respectively. On day 18, treatments with TMSC injection appeared to inhibit the DSS-induced increased mRNAs levels of *IL-1β* and *IL-6*, but without statistical significance. The level of *TNF-α* mRNA was not much altered compared with that on day 6. Interestingly, the level of *IL-17* mRNA was significantly reduced and not statistically different from the control under all treatment conditions. At the end of the experiment (on day 31), similar patterns of altered mRNA levels by all treatments were also found as those of day 18, but with some exceptions; TMSC[x4] injection statistically significantly inhibited the DSS-induced increased mRNA levels of *IL-1β* and *IL-6* by ~38% and ~54%, respectively. Interestingly, the mRNA levels of anti-inflammatory cytokines, *IL-10*, *IL-11* and *IL-13*, were not statistically different from the control under our experimental conditions (Fig 8B).

## Discussion

MSC are considered a promising medical alternative for treatment of various inflammatory and autoimmune diseases since they have been shown to have the capability of targeting injured tissues and secreting paracrine factors for anti-inflammation [34]. Although well-known MSC isolated from bone marrow and adipose tissues have been tested to treat colitis due to their anti-inflammatory properties, a highly invasive procedure is required to obtain cells from adult donors. Previously, we reported the noninvasive isolation of TMSC from human palatine tonsillar tissues. The capacity of both high yield and rapid growth of TMSC provides more secure and feasible MSC therapy for clinical applications. Of course, several studies also highlighted that TMSC are capable of inducing immunosuppression, suggesting that TMSC are suitable for the treatment of inflammatory and autoimmune diseases. In this study, we tested whether TMSC have therapeutic potential in a murine model of colitis, a chronic disorder related to immune dysfunction, and found that multiple intraperitoneal injections of TMSC clearly attenuate DSS-induced chronic colitis in mice.

The most important finding in this study was that the intraperitoneal injection of TMSC alleviated DSS-induced chronic colitis, in particular when TMSC are administered multiple times. Although injection with TMSC[x2] also exhibited some advantages, in this study, injection with TMSC[x4] yielded statistically greater therapeutic effects based on several assays. This dose-dependent increase in therapeutic effects of TMSC on colitis are largely consistent with results from a previous study using murine BM-MSC in an experimental murine colitis model [35]. However, at the beginning of this study, injection with a double dose of TMSC (i.e., 2 x 10^6^ cells) twice did not increase the effect of TMSC on DAI scores compared to a single dose (i.e., 1 x 10^6^ TMSC) twice (S1 Fig). Furthermore, unexpectedly DAI scores increased in colitis mice injected with 5 x 10^6^ TMSC twice. These results suggest that the number of injected cells is not the only factor regulating TMSC effects on colitis treatment. Establishing the optimal volume or injection number of TMSC will help to maximize the therapeutic effects and avoid potential adverse events occurred in host tissues such as hypoxia, poor nutrition and inflammatory reactions.

A previous study reported that intraperitoneally injected MSC form aggregates [36]. In this regard, one recent study demonstrated that *in vitro* three-dimensional (3D) spheroid culture of murine BM-MSC enhanced the efficiency of MSC to attenuate experimental colitis [35]. Based on these results, it is now anticipated that constructing TMSC spheroids may increase their beneficial effects on colitis. It is well known that when mesenchymal stem cells are cultured in 3D culture system, they promote cell differentiation [37, 38]. Most recently, we reported that implantation with self-assembled 3D TMSC spheroids led to favorable effects on survival rates and parathyroid hormone levels in parathyroidectomized rats compared to plain 2D TMSC culture [25]. Because of the higher levels of CD31, an endothelial cell marker, and N-cadherin, a protein responsible for cell-to-cell interaction, in the skin area of implanted TMSC spheroids from our previous study, we addressed whether the improved efficiency by spheroid implantation was attributable in part to increased TMSC viability in host tissues. Recently, it has been reported that mesenchymal stem cell transplantation using scaffold is effective in inhibiting inflammation [39, 40]. Therefore, further study on the effect of TMSC on the colitis model in the case of three-dimensional culture or two-dimensional culture will be further investigated.

As expected, injection with TMSC[x4] significantly improved DSS-induced body weight loss, colon length shortening and DAI score. In particular, at day 31, DSS-induced weight loss was almost prevented (Fig 4B) and DAI score in TMSC[x4]-treated group was almost reduced to the level of control (Fig 5). Likewise, we also showed that *IL-1β* and *IL-6* gene expression were significantly down-regulated at day 31 in experimental colitis mice when injected with TMSC[x4]. IL-1β is a member of the IL-1 family, is produced by activated monocytes and macrophages, and is an important mediator of inflammatory responses. IL-1β in inflamed intestine is mainly produced by infiltrating lamina propria monocytes in the IBD mucosa [41]. Furthermore, IL-1β activity in colitis is correlated with the severity of the disease [42]. IL-6 also plays an important role in IBD and is clinically relevant in uncontrolled chronic inflammation of the gastrointestinal tract [43]. Accordingly, the decrease in these two genes associated with TMSC[x4] may provide a target mechanism by which TMSC therapy could alleviate DSS-induced colitis development. Although several other pro-inflammatory cytokines, such as IL-17 and TNF-α, have also been reported to be associated with IBD development [44, 45], our data did not support such findings under the conditions tested. In addition to pro-inflammatory cytokines, anti-inflammatory cytokines were also reported to be associated with IBD development; among these anti-inflammatory cytokines, IL-10 is a particularly interesting cytokine involved in IBD because the knock-out of the *IL-10* gene in mouse results in manifesting intestinal inflammation which resembles IBD [46]. Under our experimental conditions, however, all the anti-inflammatory cytokines tested, including IL-10, are unlikely to be associated with DSS-induced IBD development (Fig 8B). Although the reasons underlying little association between anti-inflammatory cytokines and IBD are yet to be identified, it is noted that alterations in cytokine expression were controlled by many factors, such as the type of stimulants and duration of stimulation, as well as the type of cells. In this regard, a previous study reported that IL-10 therapy had no effect in the indomethacin-induced bowel inflammation rat model [47]. Furthermore, a systematic review analyzed from data of randomized clinical trials also showed no statistically significant differences between IL-10 therapy and placebo for complete or clinical remission in Crohn’s disease, a type of IBD [48].

Interestingly, we unexpectedly found that the injection with TMSC[x4] did not inhibit the histologic changes induced by DSS. Several previous reports showed histopathological improvements particularly at the distal colon [33, 49], which were not reproduced in our study, implying that the beneficial effects of TMSC injection on colitis may not be associated with improved histology. In some respects, however, our findings do agree with the most recent study, which showed that direct intraluminal injection with murine BM-MSC spheroids did not significantly decrease mucosal lesions in the distal colon or endoscopy score, although it significantly alleviated DSS-induced colitis, as evidenced by body weight and DAI scores [35]. Furthermore, our current data also reproduce those from human IBD; clinical remission by definition cannot be solely explained by histopathological remission, as evidenced in one clinical study showing that most of patients with IBD exhibited mucosal inflammation despite clinical remission [50]. To resolve this, we must clarify the discrepancy between the substantial recovery of several well-known surrogate markers including DAI scores for colitis and the lack of improvement in histopathological markers.

In conclusion, based on several advantages of TMSC over other MSC in clinical use, the current study demonstrates that multiple injections of TMSC are more clinically feasible, providing a favorable strategy for treatment of colitis and other diseases involving destructive inflammation.

## Supporting information

S1 Fig Disease activity index (DAI) scores in experimental colitis mice with respect to various doses of TMSC treated. Colitis mice were established by receiving 1.5% DSS and phosphate-buffered saline (DSS+PBS). Colitis mice were then treated with TMSC twice as described in the text. In this particular experiments, three different doses of TMSC (i.e., 1x10^6^, 2x10^6^, and 5x10^6^ cells per injection) were injected into colitis mice, and named DSS+TMSC[1x10^6^ cells], DSS+TMSC[2x10^6^ cells], and DSS+TMSC[5x10^6^ cells], respectively. Control mice did not received DSS (Normal). Scores of DAI were measured at day 31. Injection with TMSC twice showed significantly decreased DAI scores. However, the increasing doses of TMSC did not further decrease in DAI scores.
(PDF)
