# Supplementary material for: Therapeutic potential of tonsil-derived mesenchymal stem cells in dextran sulfate sodium-induced experimental murine colitis
Source: PLoS One. 2017 Aug 30;12(8):e0183141. doi: 10.1371/journal.pone.0183141 (PMC5576698; doi:10.1371/journal.pone.0183141)
Supplement: S1 Fig — Colitis mice were established by receiving 1.5% DSS and phosphate-buffered saline (DSS+PBS). Colitis mice were then treated with TMSC twice as described in the text. In this particular experiments, three different doses of TMSC (i.e., 1x10^6^, 2x10^6^, and 5x10^6^ cells per injection) were injected into colitis mice, and named DSS+TMSC[1x10^6^ cells], DSS+TMSC[2x10^6^ cells], and DSS+TMSC[5x10^6^ cells], respectively. Control mice did not received DSS (Normal). Scores of DAI were measured at day 31. Injection with TMSC twice showed significantly decreased DAI scores. However, the increasing doses of TMSC did not further decrease in DAI scores. (PDF) [file pone.0183141.s001.pdf]

Supporting Information

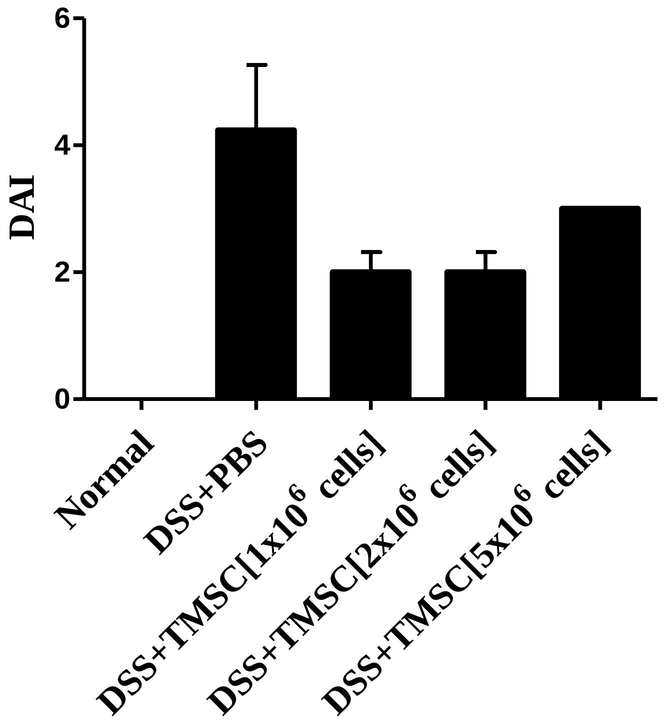

S1 Fig. Disease activity index (DAI) scores in experimental colitis mice with respect to various doses of TMSC treated. Colitis mice were established by receiving 1.5% DSS and phosphate-buffered saline (DSS+PBS). Colitis mice were then treated with TMSC twice as described in the text. In this particular experiments, three different doses of TMSC (i.e., $1x10^6$, $2x10^6$, and $5x10^6$ cells per injection) were injected into colitis mice, and named DSS+TMSC[$1x10^6$ cells], DSS+TMSC[$2x10^6$ cells], and DSS+TMSC[$5x10^6$ cells], respectively. Control mice did not received DSS (Normal). Scores of DAI were measured at day 31. Injection with TMSC twice showed significantly decreased DAI scores. However, the increasing doses of TMSC did not further decrease in DAI scores.
